# Sulfur-Doped and Bio-Resin-Derived Hard Carbon@rGO Composites as Sustainable Anodes for Lithium-Ion Batteries

**DOI:** 10.3389/fchem.2020.00241

**Published:** 2020-04-02

**Authors:** Qinyuan Huang, Jinbo Hu, Shujing Wen, Xiang Zhang, Gonggang Liu, Shanshan Chang, Yuan Liu

**Affiliations:** College of Materials Science and Engineering, Central South University of Forestry and Technology, Changsha, China

**Keywords:** bio-resin-derived hard carbon, tannin–furanic resins, sulfur-doped, rGO, lithium-ion battery

## Abstract

Hard carbon derived from fossil products is widely used as anode material for lithium-ion batteries. However, there are still several main shortcomings such as high cost, and poor rate performance, which restrict its wide application. Then tremendous efforts have been devoted to developing biomaterials in the battery applications. Recently, especially agricultural and industrial by-products have attracted much attention due to the electric double-layer capacitors. Herein, we report the sulfur-doped hard carbon (SHC) materials from the tannin-furanic resins (TF-Resin) of the derived agricultural by-products, followed by enveloping rGO on its surface through the hexadecyl trimethyl ammonium bromide. SHC provides sites for the storage of lithium, while the rGO layers can offer a highly conductive matrix to achieve good contact between particles and promote the diffusion and transport of ions and electrons. As a result, the SHC@rGO shows excellent lithium storage performance with initial discharge capacity around 746 mAh g^−1^ at a current density of 50 mA g^−1^, and shows superb stability keeping capacity retention of 91.9% after 200 cycles. Moreover, even at a high current density of 2,000 mAg^−1^, SHC@rGO still delivers a specific capacity of 188 mAg^−1^. These desired promising properties are active to the implement in the possible practical application.

## Introduction

Nowadays, lithium-ion batteries (LIBs) have been widely used with energy storage systems and portable digital devices because of their long cycle stability and high energy density (Etacheri et al., 2011; Wang et al., 2014). With a high demand for LIBs, the preparation of low-cost, environmentally friendly, and high-performance anode materials has been substantially researched in recent years (Liao et al., 2012). Various carbonaceous materials, such as graphitic carbon (Funabiki et al., 1999; Song et al., 2011), amorphous carbon (hard carbon) (Zhang et al., 2015, 2016, 2017) and soft carbon (Chae et al., 2014; Huang et al., 2018; Wang et al., 2018)), nanostructured carbon (graphene) (Raccichini et al., 2016; Ferguson et al., 2017), and carbon nanotubes (Liu et al., 2018; Yuan et al., 2018) have been widely investigated for their LIBs applications. Among all the anode materials for LIBs, graphitic material is the most commercially unitized because of its low cost, low potential (≈0.2 V vs. Li/Li^+^), and optimal electrical conductivity (Han et al., 2015). Nevertheless, graphite anode is far from meeting the demands for high energy/power density as a result of its limited capacity (372 mAh g^−1^) and inferior rate performance (Ge et al., 2018).

Alternatively, majority of researchers have taken notice of hard carbon because of its high specific capacity (740 mAh g^−1^) (Fey and Chen, 2001). As well-known, the precursors for preparing hard carbon are mainly petrochemical raw materials, such as a phenolic resin (Liu et al., 1996), high molecular polymers (Piotrowska et al., 2013), asphalt (Larcher et al., 1999; Mochida et al., 2001), etc. These raw materials are non-renewable substances, and the price is volatile because of fluctuations in international oil prices. Also, carbonization of these products often causes environmental issue such as the release of formaldehyde or other toxic carcinogens. Fortunately, biomass is a rich and renewable potentially green material. A large amount of biomass with little or no economic value can be used as a cheap and effective source of carbon precursors to produce materials that are environmentally and economically of high added value. Most natural resources have been reported including bamboo chopsticks (Jiang et al., 2014), wood (Zhang et al., 2019), soybean (Xu et al., 2015), wheat flour (Lim et al., 2017), corn cob (Liu et al., 2016), peanut shells (Ding et al., 2015), cherry stones (Arrebola et al., 2010), silk (Hou et al., 2015), coconut oil (Gaddam et al., 2016), and mangrove (Liu et al., 2010), were widely used as electrodes for energy storage applications. However, the lower conductivity of hard carbon than that of graphitizable carbons is another defect, which results in poor rate performance. In order to tackle these problems, great efforts have been made to adjust the surface structure of hard carbon, such as the construction of hybrid anodes (Guo et al., 2008), thermal carbon coatings (Ohzawa et al., 2005; Lee et al., 2007), and vacuum and oxidation treatments (Fujimoto et al., 2010; Liu et al., 2010). Although these surface modification methods may improve the coulombic efficiency of hard carbon, their rate performance and cyclability are far from satisfactory. Moreover, the further modification optimization of hard carbon materials is quite meaningful for practical application.

Corn cob and wood bark are abandoned agricultural byproducts and are generally burnt, which not only leads to air pollution but also wastes resources. To date, tannin–furanic resins have received great attention because of tannin and furfural alcohol, which are extracted from wood bark and corn cob (Tondi and Pizzi, 2009; Meikleham and Pizzi, 2010). Thus far, the synthesis of resins without formaldehyde has been an attractive focus (Tondi et al., 2009; Li et al., 2013). With the development of technology, possible applications of the natural tannin–furanic resin were recently presented (Tondi et al., 2016). It is active that the epoxy novolac resin as the hard carbon source and coated rGO for LIBs have shown the outstanding stability and rate capability due to via constructing the conductive network (Zhang et al., 2015). Herein, we report the synthesis of SHC materials derived from the biomass TF-resin with the carbonization method and enveloped with the rGO on its surface through the hexadecyl trimethyl ammonium bromide. SHC provides active sites for the storage of lithium, whereas the rGO layer can offer a highly conductive matrix to achieve contact between particles and promote the diffusion and transport of ions and electrons. As a result, the SHC@rGO shows excellent lithium storage performance with an initial discharge capacity of approximately 746 mAh g^−1^ at a current density of 50 mA g^−1^, and shows superb stability keeping a capacity retention of 91.9% after 200 cycles. Furthermore, even at a high current density of 2,000 mA g^−1^, SHC@rGO still delivers specific capacity of 188 mA g^−1^. This study compares the following petrochemical raw materials: (i) tannins and furfuryl alcohol raw materials, which have the advantages of environmental friendliness, low-cost, and renewability; (ii) the full biomass-derived hard carbon, which has optimal electrochemical properties; and (iii) it provides a reference for the application of the full biomass resin materials in energy storage materials.

## Experimental Section

Tannin extract (60 g), furfuryl alcohol (180 g), 100 mL deionized water, and 8 mL p-toluene sulfonic acid (65 wt%), which acted as the hardener, were mixed at room temperature. The precursor was treated at 60°C for 1 h in a rotary evaporator and then cured in the drying box at 60, 100, and 150°C for 2, 1, and 24 h, respectively. The cured precursor was heated at 500°C for 1 h with 3°C min^−1^ and then ground to powders after cooling under the argon atmosphere. Finally, the sample was heated at 1,000°C under the argon flow for 1 h with a ramp rate of 10°C min^−1^ to prepare the SHC.

SHC (2 g) and GO (0.4 g) were sonicated for 0.5 h in 120 mL (20 wt%) ethanol to disperse GO sheets and SHC powders. The 0.1 g hexadecyl trimethyl ammonium bromide was dissolved in the resulting suspended liquid through magnetic stirring for 5 h at room temperature. The suspension was vacuum filtered, dried in air at 100°C, and then heated to 1,000°C under argon flow for 1 h at a heating rate of 10°C min^−1^ in a vacuum tube furnace to prepare the SHC@rGO.

## Material Characterization

The morphologies of the sample were investigated with a scanning electron microscope (SEM). The morphology and microstructure of the SHC@rGO sample were characterized by transmission electron microscopy (TEM F20) and scanning transmission electron microscopy equipped with an energy dispersive spectrometer (EDS). The X-ray photoelectron spectra (XPS) were obtained by a Thermo Fisher Scientific ESCALAB 250Xi spectrometer. X-ray diffraction (XRD) was recorded on an XD-2X X-ray diffractometer with Cu–Kα radiation (λ = 1.5406 A, 30 KV, 20 mA) at the scan rate of 8°C min^−1^. Raman spectra were collected using a Raman spectrometer (Alpha300-R) with 532 nm. Nitrogen adsorption and desorption isotherms were determined through nitrogen physisorption on a Micro Active for an ASAP 2460 analyzer. Comprehensive thermal analysis (TG-DSC) was obtained using a SETARAM AETSYS-24 comprehensive thermal analysis instrument, starting from room temperature to 1,200°C at a heating rate of 10°C min^−1^ under an N_2_ gas atmosphere.

## Electrochemical Testing

Electrochemical measurements were performed using a 2016 coin-type battery using lithium metal as the anode. The working electrode was composed of active materials (SHC and SHC@rGO), the conductivity material (super P), and the binder (PVDF) in a weight ratio of 8:1:1 dissolved in N-methyl pyrrolidinone. The slurry was then spread evenly on the copper foil and dried in a vacuum oven at 120°C for 12 h. After drying, the electrode was cut into a disc with a diameter of 12 mm. The carrying mass of SHC@rGO on the anode is 0.55–0.66 mg·cm^−2^. The mass loads of rGO in SHC@rGO is 0.11–0.13 mg·cm^−2^. A solution of 1 M LiPF_6_ in ethylene carbonate, diethyl carbonate, and dimethyl carbonate (1:1:1 by volume ratio) was used as an electrolyte, and a polypropylene film was used as a separator. The cycling and rate performance of SHC and SHC@rGO were tested on a land battery test system at 25°C. Cyclic voltammetry (CV) and electrochemical impedance spectroscopy (EIS) measurements were performed on an Ivium electrochemical workstation.

## Results and Discussion

**Figure 1:**
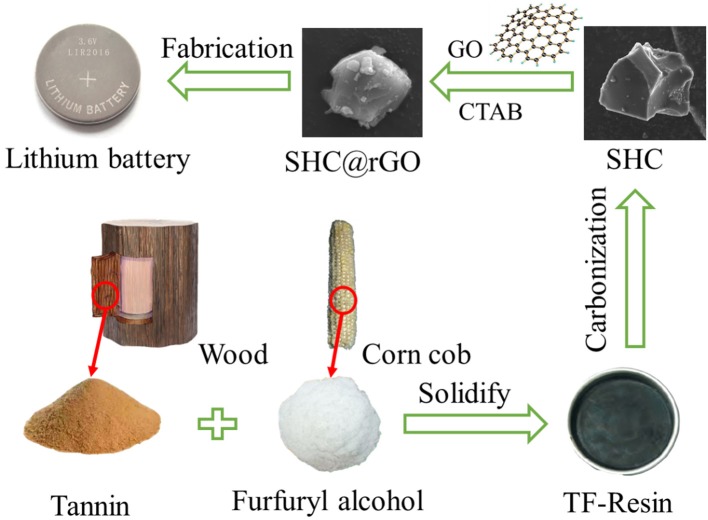
Schematic illustration of the preparation of SHC and SHC@rGO.

Figure 1 shows the schematic illustration for the synthesis of SHC and SHC@rGO materials derived from the biomass TF-Resin. Firstly, TF-Resin precursors were obtained a simple solidify method (see Experimental Section for detail). Subsequently, the obtained TF-Resin precursors were calcinated under Ar atmosphere to prepare SHC. Finally, the SHC@rGO was obtained after enveloped with the rGO on its surface through the hexadecyl trimethyl ammonium bromide and assembled into a 2016 Coin battery.

**Figure 2:**
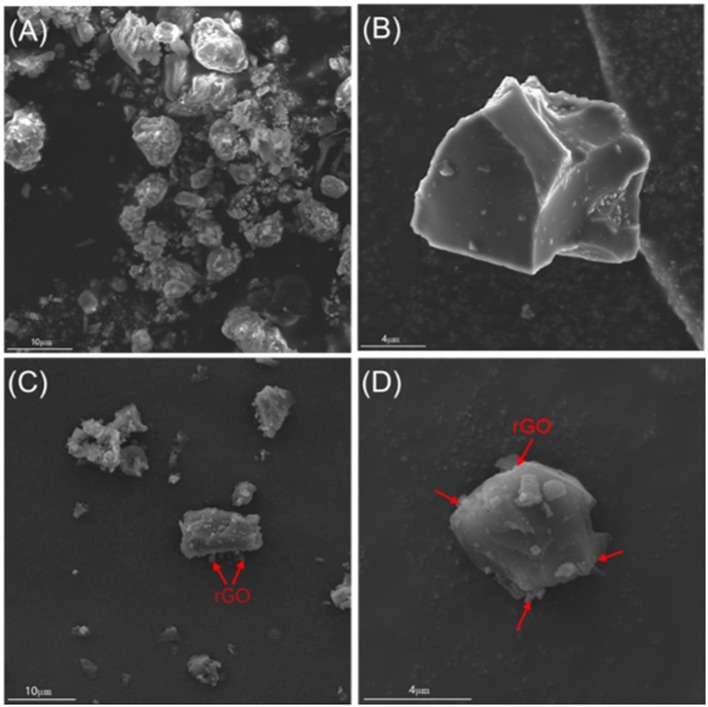
SEM images of SHC **(A,B)** and SHC@rGO **(C,D)** composites.

Figure 2 shows the SEM images of SHC and SHC@rGO. It can be seen that SHC sample presents irregular morphology and the surface is non-porosity and glossy (Figures 2A,B). The SHC@rGO sample consists of irregular HC particles and lamellar reduced graphene oxide (rGO) with a wrinkled surface (Figures 1C,D). which is help construct the conductive network and guaranteeing fast electron conduction.

**Figure 3:**
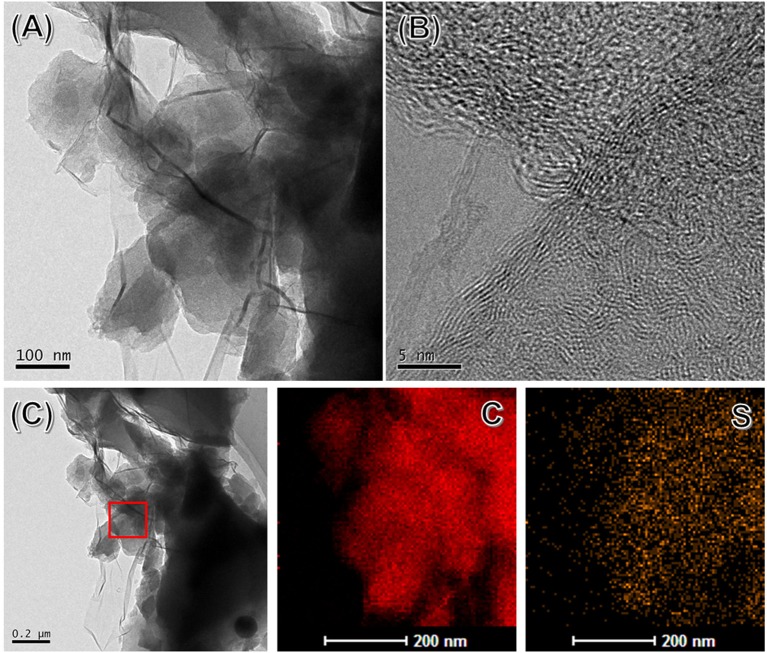
A TEM **(A)**, HRTEM image **(B)**, and EDS elemental mapping **(C)** of SHC@rGO.

The microstructure of SHC@rGO composites was further studied by TEM and high-resolution TEM (HRTEM). As presented in Figure 3A, the SHC is partially wrapped by corrugated graphene nanosheets. It can be known that winkled rGO is well bonded to SHC particles due to van der Waals force between graphene sheets and SHC, which is beneficial to transport electrons and lithium ions. HRTEM image of SHC@rGO sample taken on the edges of rGO is shown in Figure 3B. The rGO consists of several stacked layers and its thickness is <5 nm. Graphene coating can effectively promote the transmission rate of electrons and ions and maintain the integrity of the conductive network, which contribute to improved electrochemical performance. The EDS mapping of SHC is shown in Figure 3C. The EDS element mapping of C and S confirms that the S element is evenly distributed throughout the hard carbon.

**Figure 4:**
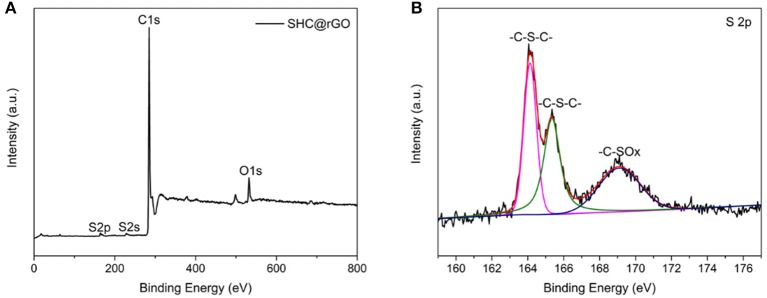
XPS spectra of SHC@rGO composite and survey spectrum **(A)** and S 2p spectra **(B)**.

To determine the doping level and bonding configuration of S, we performed the XPS analyzation of SHC@rGO. As presented in Figure 4A, the predominant peaks at 165.0, 228.0, 285.0, 402.0, and 532.0 eV are assigned to S 2p, S 2s, C 1s, N 1s, and O 1s, respectively. The S 2p spectra (Figure 4B) showed three peaks at 164.1, 165.3, and 169.2 eV. The former two peaks are assigned to the thiophene-S groups (–C–S–C–), and the third peak corresponds to the –C–SOx groups (*X* = 2–4) (Choi et al., 2011; Kiani et al., 2019). The XPS results indicate that the S atom has been chemically bonded to the SHC.

**Figure 5:**
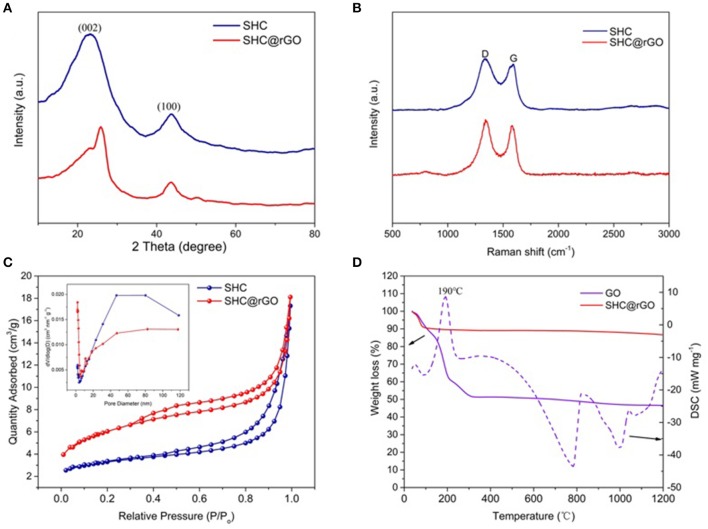
**(A)** XRD patterns of SHC and SHC@rGO, **(B)** Raman spectra of SHC and SHC@rGO, **(C)** calculated pore size distribution and nitrogen adsorption isotherms (insert C) for SHC and SHC@rGO, and **(D)** TGA curve of the decomposition of SHC and SHC@rGO composite (Atmosphere: N_2_, heating rate: 10°C min^−1^).

The XRD patterns of the SHC and SHC@rGO samples are presented in Figure 5A. It can be seen that the XRD patterns of SHC and SHC@rGO are both composed of wide diffraction peaks at 22°-26°and 44°-45° 2θ corresponding to (002) and (100), respectively, which are typical of carbonaceous materials with an amorphous-like structures (Inagaki et al., 2001). The *D*_002_ values of SHC and SHC@rGO are summarized in Table S1. SHC@rGO (0.351 nm) exhibits smaller average interlayer distance than SHC (0.380 nm), which is induced by rGO addition, because of the graphene oxide is reduced at high temperature to form a layered structure with decreasing interlayer spacing.

Figure 5B shows the Raman spectra of SHC and SHC@rGO. The characteristic peaks of the two samples are similar. The D-bands peak at about 1,340 cm^−1^ and the G-bands peak at about 1,590 cm^−1^. Peak D-bands and G-bands represent sp^3^ hybridization and sp^2^ hybridization, respectively. The ratio of the integrated areas of the two peaks can manifest the degree of order of carbon materials. The *I*_*D*_*/I*_*G*_ of the SHC and SHC@rGO is 1.13 and 1.08, respectively (Ferrari and Robertson, 2000). It was shown that the carbon atoms in sp^2^ hybridization increased after graphene was added, the structure order and graphitization degree of the materials increased. This result was consistent with the results of XRD.

The nitrogen adsorption-desorption isotherm test (77 K) is an important tool of characterizing the specific surface area and pore volume of porous materials. Figure 5C shows the N_2_ adsorption-desorption curve and the pore size distribution based on the BJH (Barrett-Joiner-Halenda) model of SHC and SHC@rGO. It can be seen that the N_2_ adsorption isotherm of SHC and SHC@rGO belong to the III type isotherm. The calculated pore size distributions from the adsorption branches using the density functional theory (DFT) (inset of Figure 5C) model for SHC and SHC@rGO, suggest the characteristics of mesoporosity and microporosity. The specific surface area *S*_BET_ and pore volume data are listed in Table S1. It can be seen from the table that the BET specific surface area increases from 11.21 m^2^ g^−1^ of SHC to 21.02 m^2^ g^−1^ of SHC@rGO, the pores volume remains basically comparative. For these two samples, the mesopore volume accounts for more than 90% of the total pore volume.

Figure 5D shows the TG/DSC curves for GO and SHC@rGO. The TG/DSC of pure SHC curve is not shown because it has no heat loss and exotherm. It can be seen that GO has a distinct exothermic peak at 190°C with significant thermal weight loss, which is caused by the volatilization of a large number of water molecules adsorbed on GO. The thermogravimetric curve of SHC@rGO showed a slight decrease due to the small proportion of GO in the SHC@rGO mixture. When the temperature increases from 254 to 388°C, the DSC curve of GO shows a taro peak with a slight thermal weight loss, indicating a moderate exothermic reaction, which is ascribed to escape of the large number of H atoms in GO. When the temperature is at 781 and 1,000°C, there is a significant endothermic peak with obvious thermal weight loss, the TG curve still slightly decreases, which is caused by the large amount of O atoms in GO continuously escaping at high temperature. It also shows that GO can be reduced at high temperatures. When the pyrolysis temperature is 1,200°C, the residual mass ratio of GO and SHC@rGO are 46.6 and 86.6%, respectively. Assuming that the mass of SHC does not change, the theoretical calculation shows that the residual mass ratio of SHC@rGO is 88.3%, which is basically consistent with the experimental results. It indicates that at high temperatures, most of the H and O atoms in the GO are removed.

**Figure 6:**
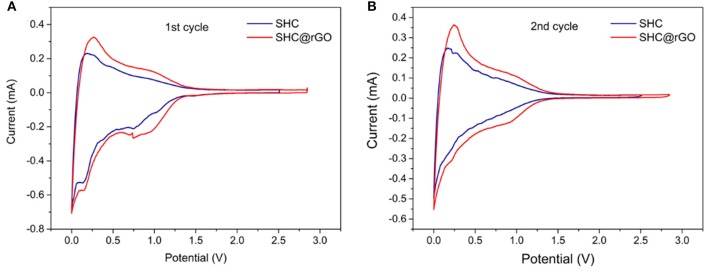
**(A)** CV curves of SHC and SHC@rGO in the first cycle and **(B)** CV curves of SHC and SHC@rGO in the second cycle.

The electrochemical profiles of SHC and SHC@rGO are characterized by coin half cells, which are composed of SHC or SHC@rGO as an anode, a lithium metal as counter electrodes, electrolyte, and a Celgard 2,400 separator. Figure 6 shows the initial two CV curves of SHC and SHC@rGO between 0 and 3 V at a sweep rate of 0.1 mV s^−1^. It can be seen that cathode reduction peaks appear at 0.70–0.75 V in the first cycle and disappear in the subsequent cycle (Figure 6B). These reduction peaks suggest that the solid electrolyte interphase (SEI) layer being formed on the surface of the carbon materials during the first lithium intercalation (Buqa et al., 2006). Cathodic current peaks reach 0.21 V because of irreversible side reactions of lithium with absorbed species or surface functional groups, a weak reduction peak was found in two samples during the first discharge process but disappeared in the second cycle (Figure 6B), which contributes to part of capacity loss leading to large irreversible capacity (Zhang et al., 2014). In addition, the anodization peak potential of SHC@rGO was lower than that of pure SHC, indicating that when graphene is added to the hard carbon material, lithium-ions are more easily deintercalated in the hard carbon material during the charge/discharge process, which will be beneficial to improve its electrochemical performance. Therefore, the graphene in SHC@rGO not only provides electronic conductivity but also improves the reversible migration ability of lithium-ions in materials.

**Figure 7:**
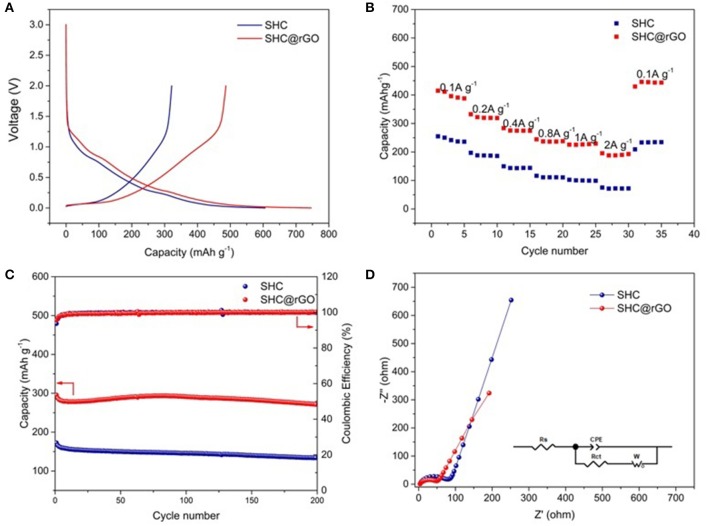
**(A)** Charge and discharge curves of SHC and SHC@rGO at a current density of 50 mA g^−1^ between 0 and 3 V, **(B)** ratio performance patterns of SHC and SHC@rGO with the charge/discharge current densities in the 100–2000 mA g^−1^ range, **(C)** cycle stability curves of SHC and SHC@rGO at 400 mA g^−1^, and **(D)** Nyquist plots of SHC and SHC@rGO at the potential of 0.1 V vs. Li/Li^+^ over the frequency range from 100 kHz to 0.01 Hz.

The initial galvanostatic discharge/charge curves of SHC and SHC@rGO at a current density of 50 mA g^−1^ are shown in Figure 7A. The charging and discharging processes correspond to the lithium removal and insertion process, respectively. It can be seen that the shape of the first charge-discharge curves of SHC and SHC@rGO is analogical, indicating that the lithium intercalation/deintercalation mechanism of SHC has not been changed after modification by wrapped GO. It can be seen that the specific capacity of SHC and SHC@rGO for the initial discharge capacities are 605 and 746 mAh g^−1^, and the charge capacities are 321 and 486 mAh g^−1^, respectively. The first irreversible specific capacity of SHC and SHC@rGO are 284 and 260 mAhg^−1^, and the initial Coulomb efficiency (ICE) of SHC and SHC@rGO are 53.1% and 65.1%, respectively. Unfortunately, the pure SHC electrodes show low ICE at the same current density, similar to previous reports about hard carbon material (Zhang et al., 2015). The battery of the low ICE is probably attributed to the following two major reasons. Primarily, the decomposition of electrolyte during the initial charge/discharge process has significantly displayed in the battery formulation of SHC derived the resin. Secondly, the formation of an irreversible solid electrolyte interface (SEI) on the surface of anode could also lead to a low ICE (Liu et al., 2016), consistent with the CV results. Nevertheless, it is active that the ICE can be improved from 53.1 to 65.1% owing to the presence of rGO in SHC@rGO. The ICE improvement of SHC@rGO was ascribed to the crucial rGO, which can construct the conductive network and promote the transport of Li^+^ ions in the electrolyte. Therefore, the SHC would be progressively modified in the prospective battery.

The rate performance of SHC and SHC@rGO prepared at different current densities are shown in Figure 7B. The rate performance of SHC@rGO is superior to that of SHC under different current densities from 0.1 A g^−1^ (410 mAh g^−1^) to 2 A g^−1^ (188 mAh g^−1^). When the current rate decreased from 2 A g^−1^ to 0.1 A g^−1^after the 25th rate cycle corresponding to discharge capacity of 444.7 mAh g^−1^. As for SHC, a much lower rate capacity was delivered at varied current densities. Both the capacity of SHC and SHC@rGO increased when the current density returned to 0.1 A g^−1^, this phenomenon can be confirmed by the electrolyte infiltration and electrode material activation during different current charge/discharge processes. Table S2 lists TF-resin prepared in this work vs. other hard carbons derived from epoxy novolac resin (Zhang et al., 2014), pitch (Kim et al., 2016), and polyphenylene sulfide (Luo et al., 2018), which demonstrates that the SHC derived from TF-resin deliver better discharge capacity and rate performance.

Cycle stability of SHC and SHC@rGO was estimated at the current density of 400 mA g^−1^ (Figure 7C). It can be inferred that from this cyclic performance, SHC and SHC@rGO delivered 134.1 and 271.8 mAh g^−1^ capacity after 200 cycles with the Coulombic efficiency near 100%, keeping capacity retention of 77.7 and 91.9%, respectively. Therefore, the SHC@rGO hybrid anode shows obviously higher cycling stability and reversible capacity, which are S-doping hard carbon provides active sites for the storage of lithium, while the rGO layers offer a highly conductive matrix and high contact area can lead to effective contact with the electrolyte into the electrode and quickly transport ions into the deeper parts of the SHC@rGO particles and graphite layer. A comparison of hard carbon prepared in this work with other biomass-derived carbons is shown in Table S3, which demonstrates that the prepared hard carbon delivers better rate capabilities and cycling performance.

The EIS measurements of SHC and SHC@rGO were tested (Figure 5D). The plots consist of a semicircle in the high-frequency region and a straight line in the low-frequency region. the straight line and the semicircle corresponding to Warburg diffusion impedance (W) and charge transfer resistance (Rct), respectively (Pan et al., 2018). Based on equivalent electric circuit, the Rct of SHC and SHC@rGO are 65.34 and 50.49Ω, respectively. The lower Rct of SHC@rGO was attributed to the presence of conductive rGO able to constructing the conductive network and facilitate the transport of Li^+^ ions, resulting in a superior electrochemical performance.

## Conclusion

In summary, biomass TF resin-derived SHC and SHC@rGO composite material was prepared as the anode material for lithium-ion battery. It is demonstrated that the rGO networks are enveloped on SHC constructing the desired microstructure. Compared with the biomass-derived pure SHC, the SHC@rGO composites show enhanced conductivity offered by rGO networks and large Li ion storage sites supported by SHC. As a consequence, SHC@rGO composite exhibits a superior electrochemical rate performance and reversible capacity. Based on this study, a new biomass derived hard carbon composites with superior electrochemical properties is reported, which is promising for low-cost and eco-friendly anode material of LIBs.

## Data Availability Statement

All datasets generated for this study are included in the article/Supplementary Material.

## Author Contributions

JH, XZ, SC, and YL: conceived and designed the experiments. QH, SW, and JH: performed the experiment. SC, JH, and XZ: supervised the work. QH, JH, and YL: wrote the paper. JH, GL, and XZ: revised the paper. JH and YL: contributed equally. All authors reviewed and approved the final manuscript.

### Conflict of Interest

The authors declare that the research was conducted in the absence of any commercial or financial relationships that could be construed as a potential conflict of interest.
