# Supplementary material for: Sulfur-Doped and Bio-Resin-Derived Hard Carbon@rGO Composites as Sustainable Anodes for Lithium-Ion Batteries
Source: Front Chem. 2020 Apr 2;8:241. doi: 10.3389/fchem.2020.00241 (PMC7142261; doi:10.3389/fchem.2020.00241)
Supplement: Supplementary file 1 [file Table_1.DOCX]

**Supporting Information**

**Sulfur-doped and bio-resin-derived hard carbon@rGO composites as sustainable anodes for lithium-ion batteries**

Qinyuan Huang^1^, Jinbo Hu^1*^, Shujing Wen^1^, Xiang Zhang^1^, Gonggang Liu ^1^, Shanshan Chang^1*^, and Yuan Liu ^1^

1College of Materials Science and Engineering, Central South University of Forestry and Technology, Changsha, Peoples Republic of China

*Corresponding author. E-mail addresses: mailto:hjb1999@hotmail.com (Jinbo Hu)

**Table S1** Structure parameters of SHC and SHC@rGO.

| Sample | d_002_(nm) | I_D_/I_G_ | S_BET_ (m²/g) | V_meso_ (cm^3^/g) |
| --- | --- | --- | --- | --- |
| SHC | 0.380 | 1.13 | 11.21 | 0.026 |
| SHC@rGO | 0.351 | 1.08 | 21.02 | 0.027 |

**Table S2** Comparison of SHC prepared in this work versus other hard carbons derived from fossil products.

| Precursors | ICE (%) | Discharge capacity (mAh g^−1^) | Discharge capacity (mAh g^−1^) |
| --- | --- | --- | --- |
| STF-resin (this work) | 53.1 | 605 (50 mA g^−1^) | 188 (2000 mA g^−1^) |
| Epoxy novolac resin | 66.7 | 585 (20 mA g^−1^) | 200 (2000 mA g^−1^) |
| Pitch | 73 | 507 (0.1 C) | 248 (5 C) |
| Polyphenylene sulfide | 99.2 | 143 (0.2C) | 81 (2 C) |

**Table S3** Comparison of hard carbon prepared in this work versus other biomass-derived carbons

| References | ICE(%) | Initial discharge capacity (mAh g^−1^) | Discharge capacity (mAh g^−1^) | Capacity retentions |
| --- | --- | --- | --- | --- |
| this work | 53.1 | 605 (0.05 A g^−1^) | 188 (2 A g^−1^) | 77.7% (400mA g^-1^,200 cycles) |
| (Gaddam et al., 2016) | 50 | 427 (0.2 A g^-1^) | 149(1 A g^−1^) | 63% (---,20 cycles) |
| (Jiang et al., 2014) | 56.6 | 230 (0.46C) | 59(12.34C) | > 100% (1C, 800 cycles) |
| ([Wang et al., 2013](#_ENREF_38)) | 49.8 | 393 (0.075 A g^−1^) | - | > 100% (75 mA g^−1^, 100 cycles) |
| (Xu et al., 2015) | - | 467.1 (0.5C) | 178(1C) | - |
| ([Zhang et al., 2019](#_ENREF_53)) | 65.7 | 624.5((0.05 A g^−1^) | 150(2 A g^-1^) | 55.2% (500 mA g^−1^, 500 cycles) |
